# Supplementary figures and images for: Kinematic gait characteristics of straight line walk in clinically sound dairy cows
Source: PLoS One. 2021 Jul 21;16(7):e0253479. doi: 10.1371/journal.pone.0253479 (PMC8294546; doi:10.1371/journal.pone.0253479)

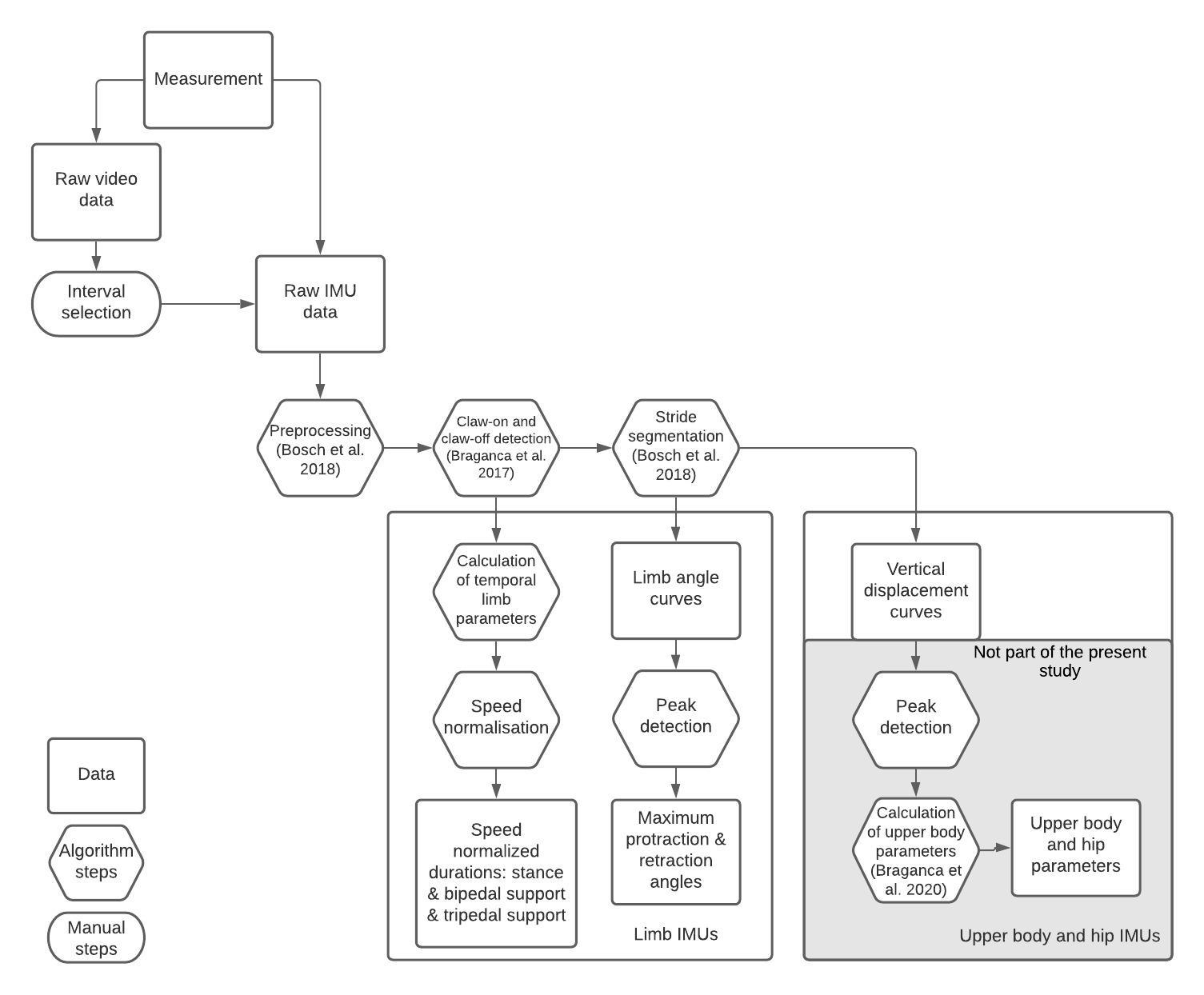

Supplement: S1 Fig — (TIF) [file pone.0253479.s001.tif]

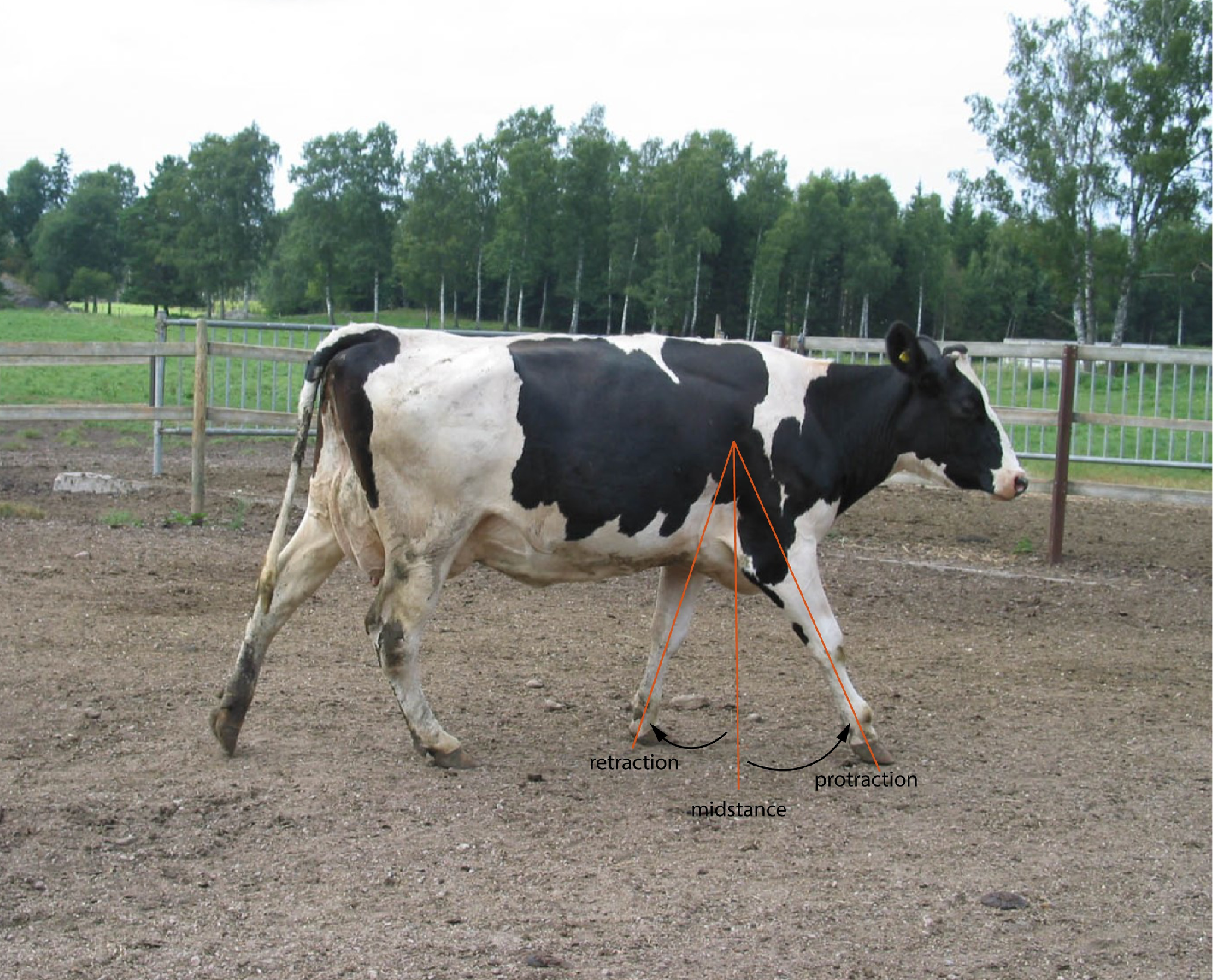

Supplement: S2 Fig — Maximal protraction is the maximal forward protraction (positive angle) from midstance and maximal retraction is the maximal backward retraction (negative angle) from midstance measured at the metacarpus/-tarsus in the sagittal plane, as adapted from horses [28, 32]. (TIF) [file pone.0253479.s002.tif]

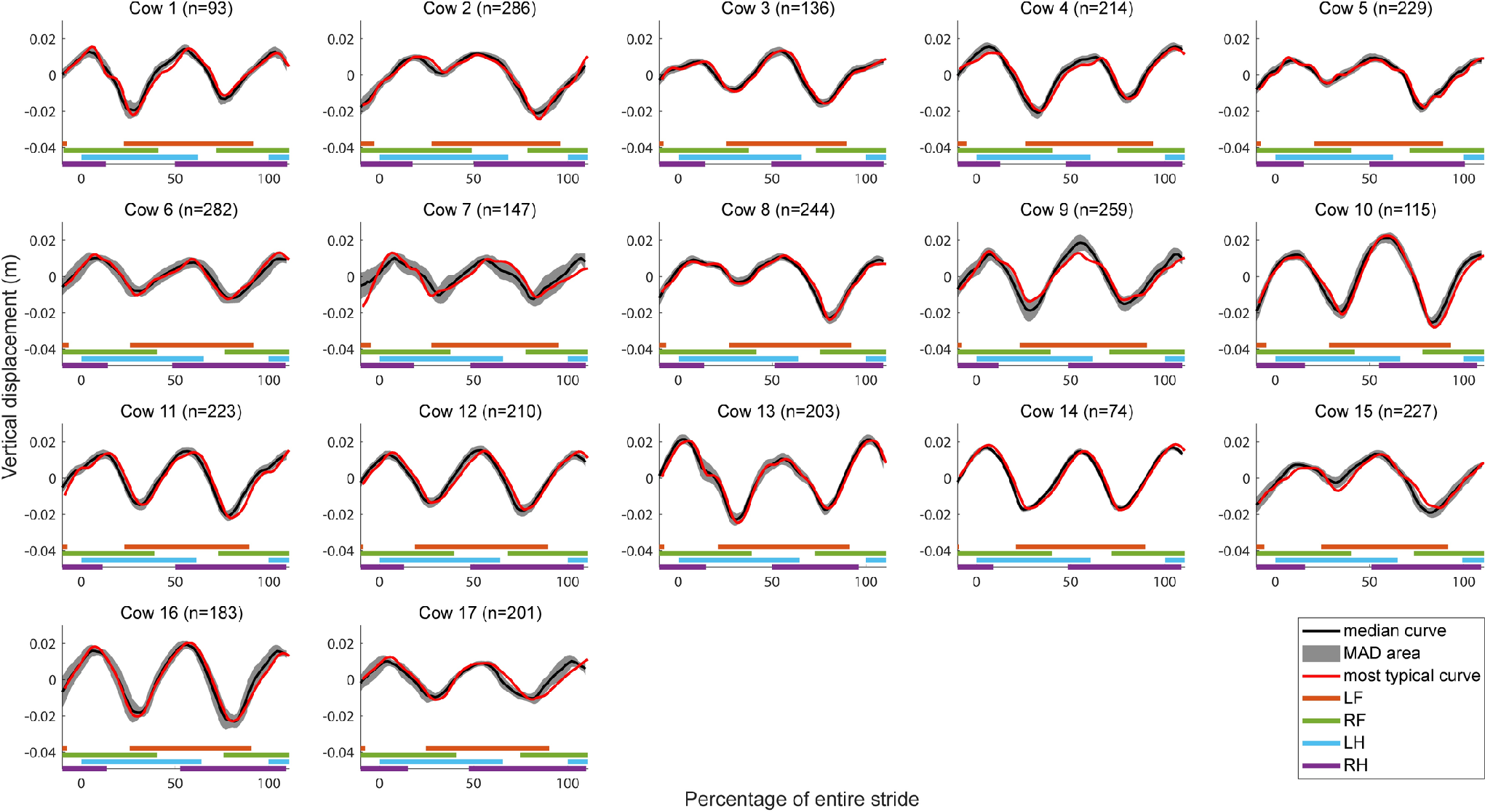

Supplement: S3 Fig — Per cow, the median curve (black), the MAD area (grey), and the most typical curve (red) is shown on a scale from zero to 100% of the entire stride duration. The stance phases of the limbs are indicated by the horizontal lines underneath the curves (orange: LF, green: RF, blue: LH, purple: RH). (TIF) [file pone.0253479.s003.tif]

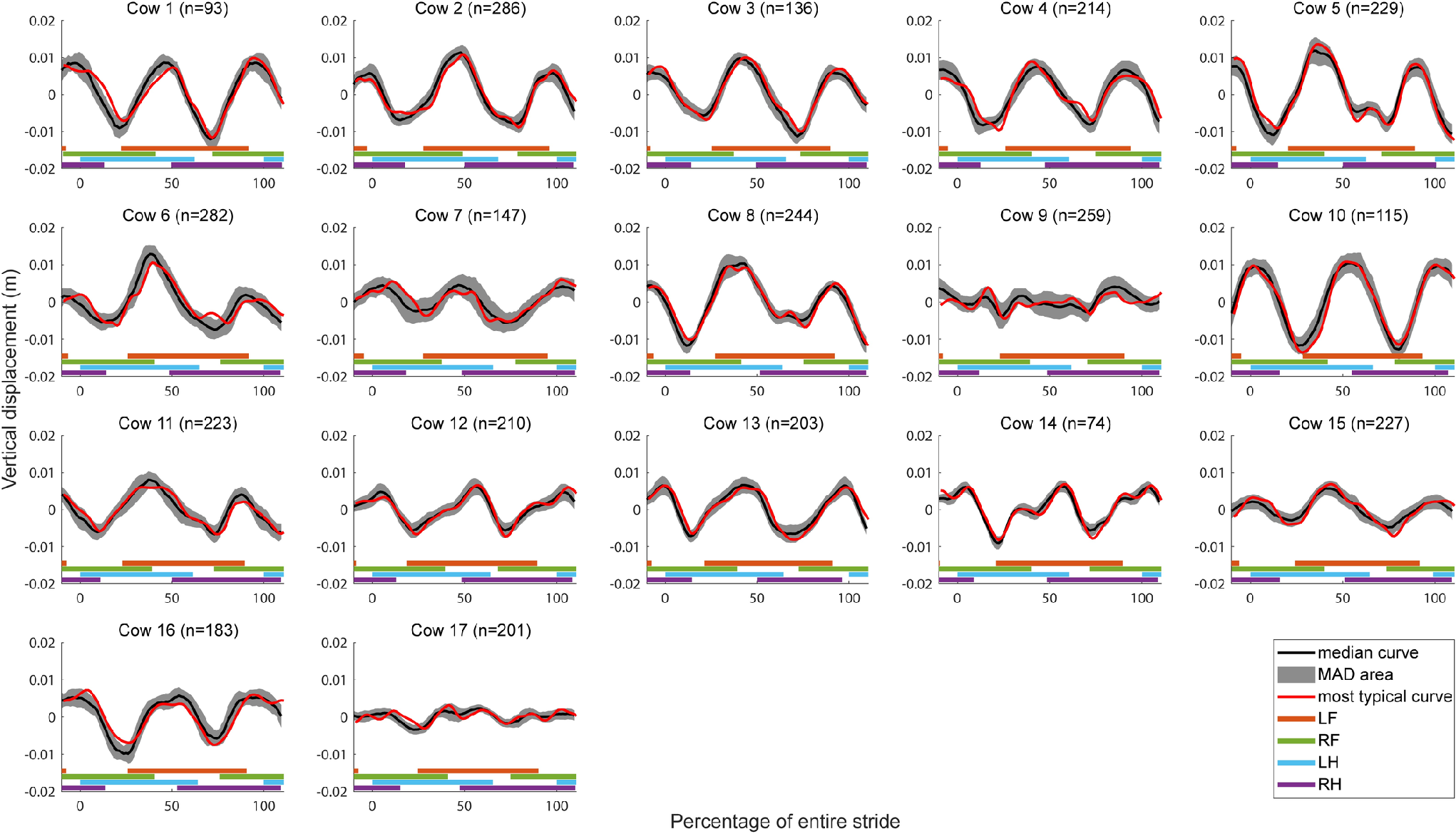

Supplement: S4 Fig — Per cow, the median curve (black), the MAD area (grey), and most typical curve (red) is shown on a scale from zero to 100% of the entire stride duration. The stance phases of the limbs are indicated by the horizontal lines underneath the curves (orange: LF, green: RF, blue: LH, purple: RH). (TIF) [file pone.0253479.s004.tif]

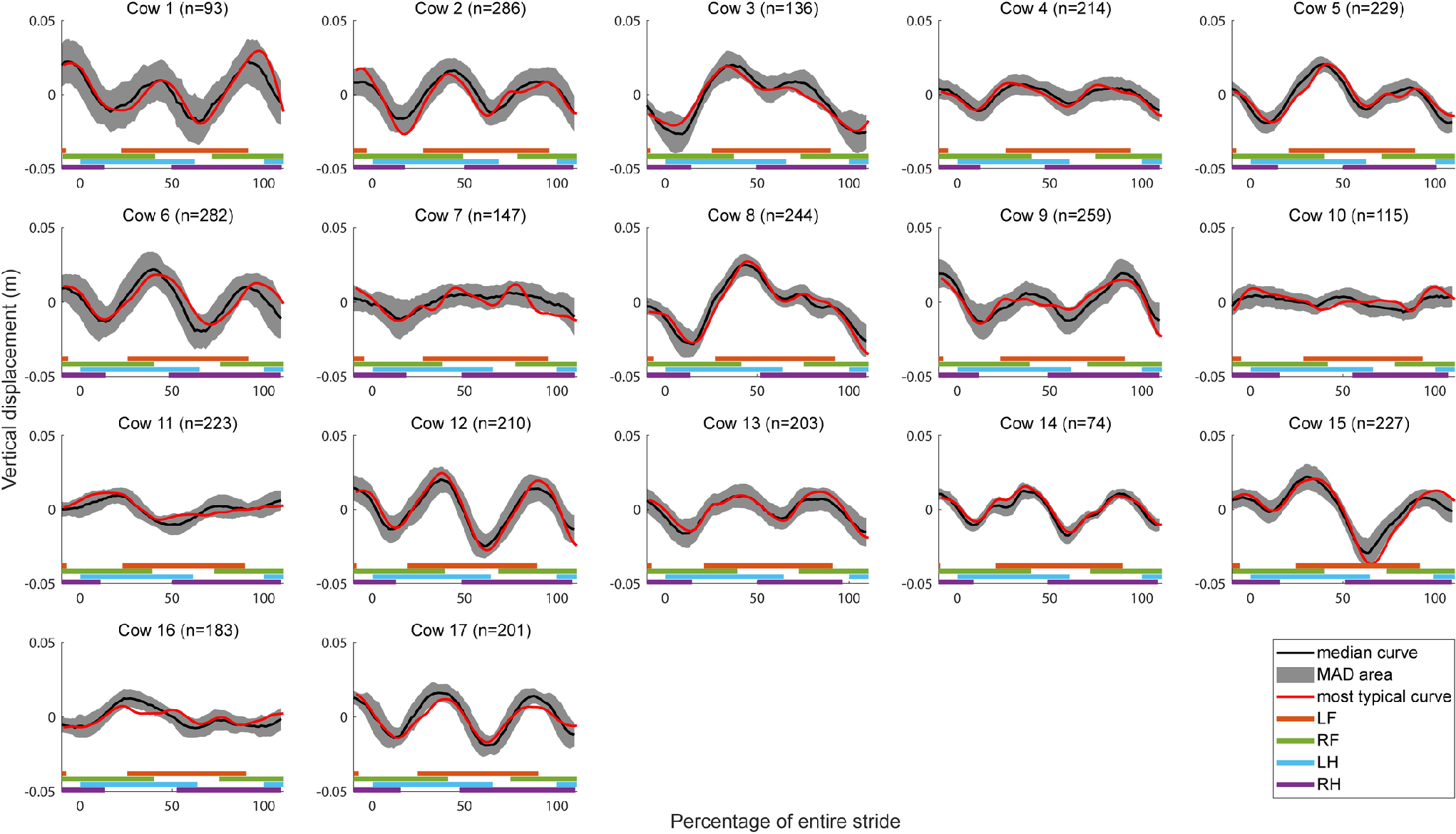

Supplement: S5 Fig — Per cow, the median curve (black), the MAD area (grey), and the most typical curve (red) is shown on a scale from zero to 100% of the entire stride duration. The stance phases of the limbs are indicated by the horizontal lines underneath the curves (orange: LF, green: RF, blue: LH, purple: RH). (TIF) [file pone.0253479.s005.tif]

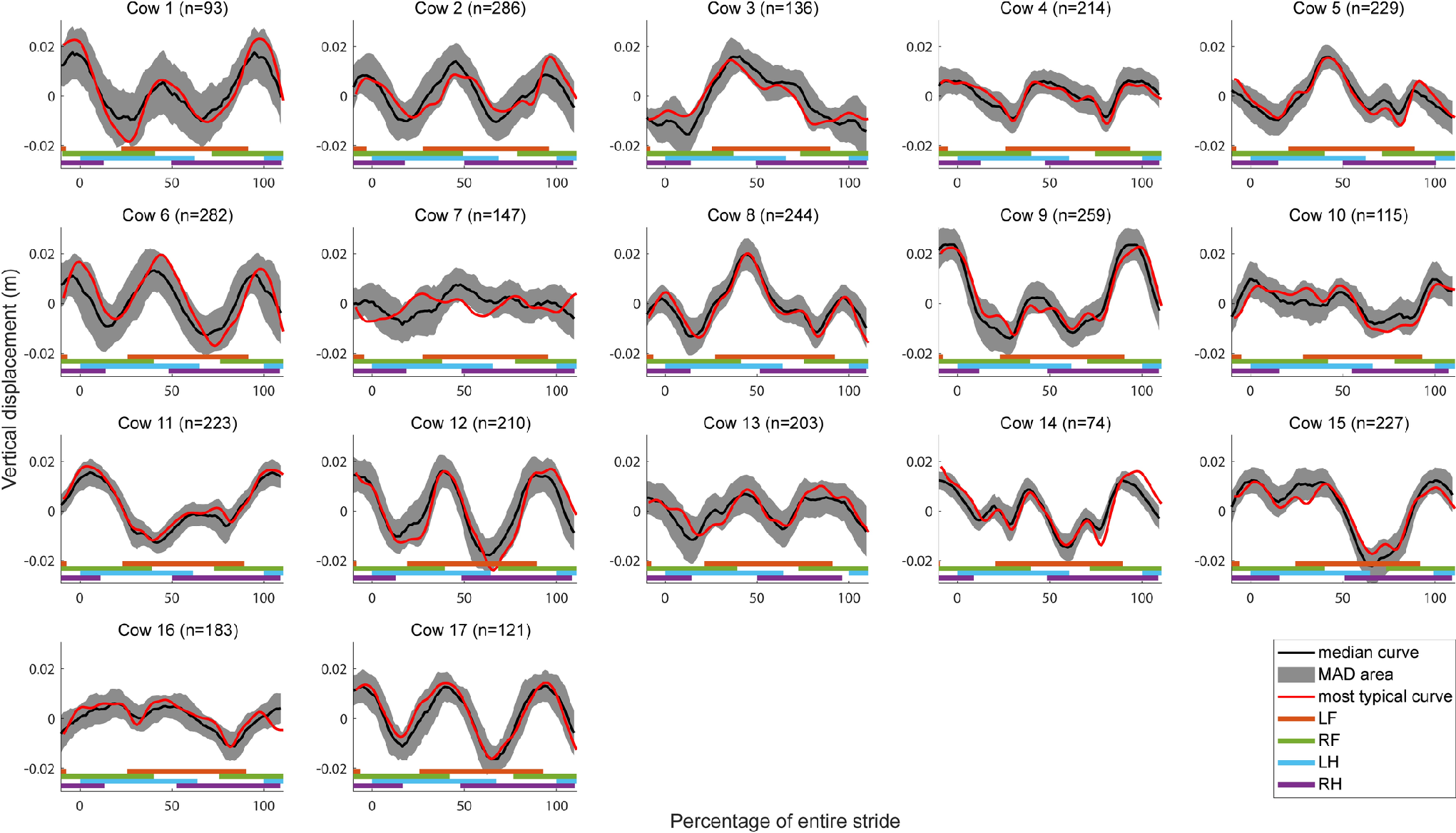

Supplement: S6 Fig — Per cow, the median curve (black), the MAD area (grey), and the most typical curve (red) is shown on a scale from zero to 100% of the entire stride duration. The stance phases of the limbs are indicated by the horizontal lines underneath the curves (orange: LF, green: RF, blue: LH, purple: RH). (TIF) [file pone.0253479.s006.tif]

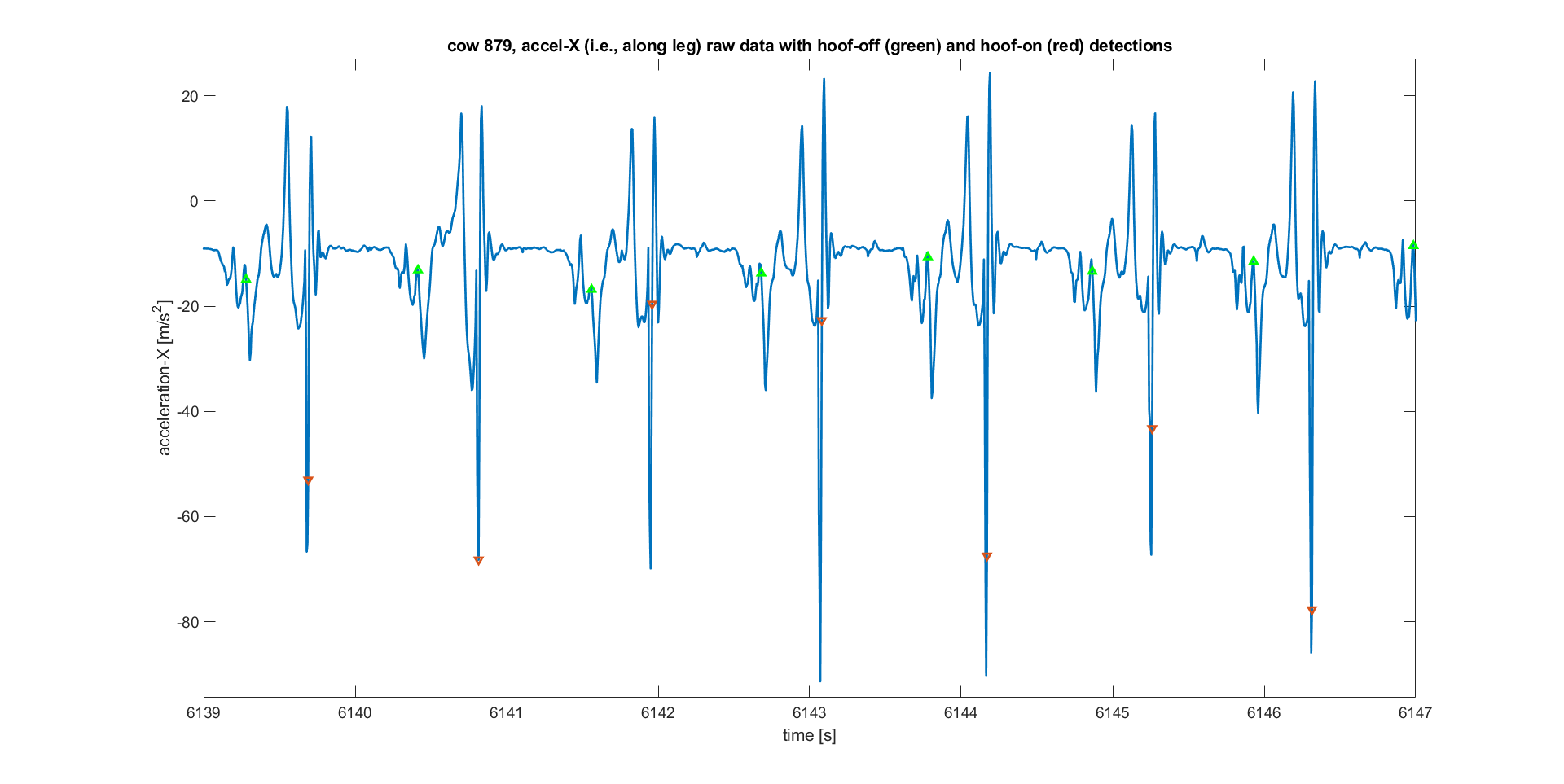

Supplement: S7 Fig — Raw acceleration data of the LF limb of cow 16 was used to show the claw-on (red) and claw-off (green) detections. (TIF) [file pone.0253479.s007.tif]

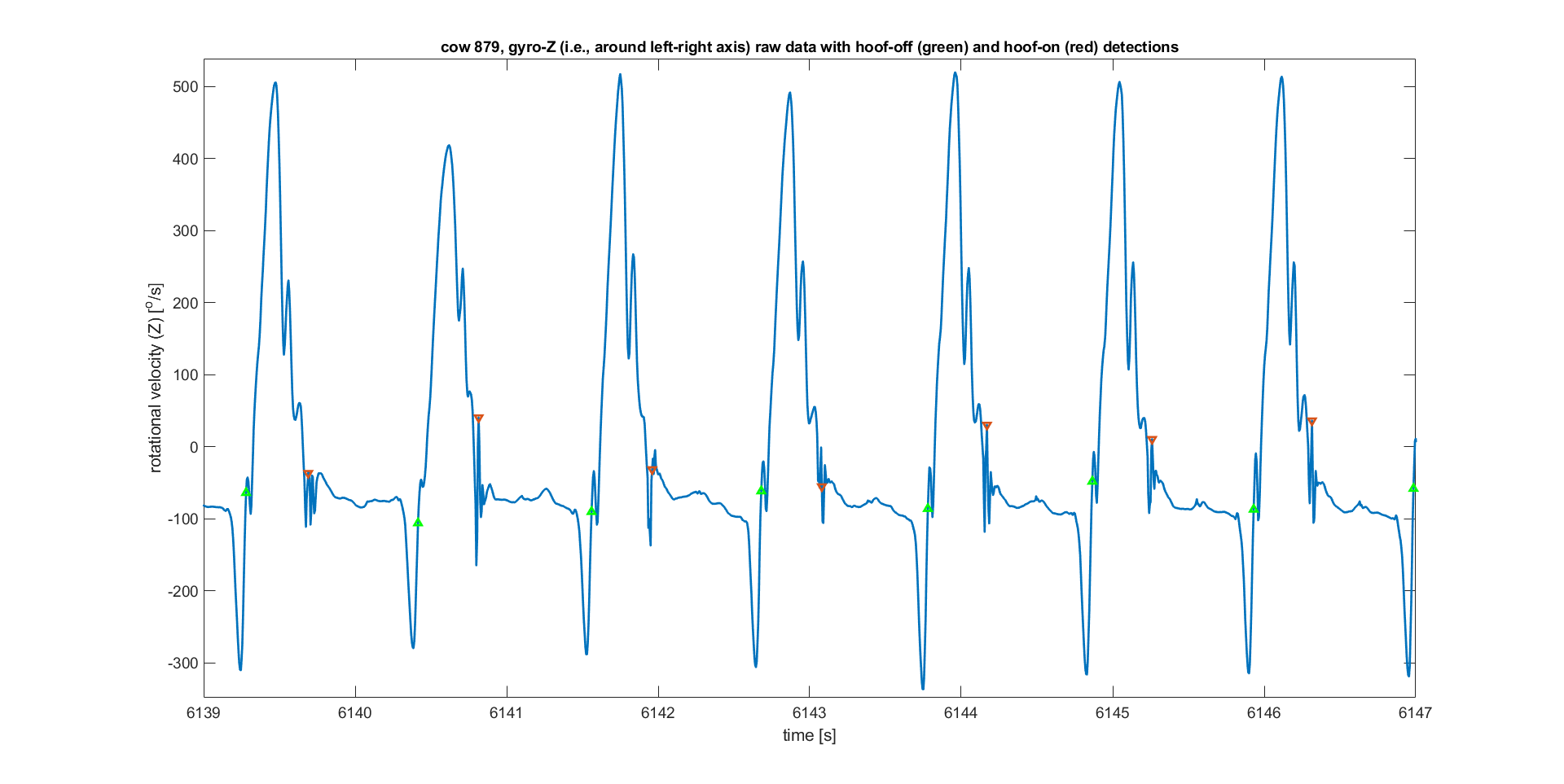

Supplement: S8 Fig — Raw gyroscope data of the LF limb of cow 16 was used to show claw-on (red) and claw-off (green) detections. (TIF) [file pone.0253479.s008.tif]
