# Supplementary material for: Kinematic gait characteristics of straight line walk in clinically sound dairy cows
Source: PLoS One. 2021 Jul 21;16(7):e0253479. doi: 10.1371/journal.pone.0253479 (PMC8294546; doi:10.1371/journal.pone.0253479)
Supplement: Attachment — Submitted filename: PONE-D-20-38036 PONE 2021.docx [file pone.0253479.s011.docx]

With interest I read your paper. The paper provides relevant data regarding using IMU sensors to detect diverse kinematics variables at multiple anatomical locations in healthy cows. Well written!

Below you find my minor comments

Abstract:

No any values available in the abstract. This makes the abstract more "descriptive"

36-38: I suggest to revise the sentence as you so far not performed your measurements in lame cows. Delete "promising" and add "maybe improve automated lameness detection" . See lines 76-78.

Keywords:

what is the difference between the pattern and characteristics?

Introduction:

40-41: add reference. Many biomechanical studies are so far performed and aimed to detect lameness in cattle.

55-56: not really. Check the pubmed. Several works have described the biomechanical gait in cattle. E.g. Flower et al. 2005

65: temporal stride parameters. Why not kinematics parameters instead of temporal parameter?

72-73: how the authors can explained the importance of applying the sensors in upper body in comparison to the "traditional" limb positions?

Materials and Methods:

84-85 were the cows guided or the halter broken and the cows walked freely. What is happen when the cows running or when there were signal artifacts of IMU?

97-89 must clearly defined. What about SU or other hoof diseases?

141: you used a low frame for you held-camera. How you performed the synchronization with IMU? It needs at least high-speed camera for this synchronization

193: may you can add the unites to the Table 1. It is not more realistic to consider the relative measurements (%) rather than the absolute values (s)?

Results and Discussion

224-225: may you can add the median

228 Figure 2 and all the figures have poor resolution so it is difficult to follow them.

Have the authors checked the differences of temporal events (e.g. stance phase duration) between left and right limbs? How are the breed, DIM, lactation numbers .. affect your results?
