# Supplementary material for: Kinematic gait characteristics of straight line walk in clinically sound dairy cows
Source: PLoS One. 2021 Jul 21;16(7):e0253479. doi: 10.1371/journal.pone.0253479 (PMC8294546; doi:10.1371/journal.pone.0253479)
Supplement: Attachment — Submitted filename: Response to reviewers.docx [file pone.0253479.s013.docx]

**Journal Requirements:**

Please review your reference list to ensure that it is complete and correct. If you have cited papers that have been retracted, please include the rationale for doing so in the manuscript text, or remove these references and replace them with relevant current references. Any changes to the reference list should be mentioned in the rebuttal letter that accompanies your revised manuscript. If you need to cite a retracted article, indicate the article’s retracted status in the References list and also include a citation and full reference for the retraction notice.

*We are not aware of referring to any retracted papers.*

**Reviewers' comments:**

*Dear reviewers, we would like to thank you for going through the manuscript again. It really improved the manuscript and we wrote down the revisions made accordingly.*

Reviewer #1: Dear authors,

First my apologies for my late review, some circumstances made me have to go off grid for a few weeks. Nevertheless, below follows my (few) remaining comments.

Second, thank you for addressing my previous comments. You did a great job providing me with detailed answers to my questions and concerns, which clarified for me most things. And I appreciate that “biomechanics” were toned down to “kinematic”, which is more to the point concerning the IMU’s.

However, some of the clarifications from the rebuttal letter were not included in your manuscript. And this (type of discussion) is exactly what I think is lacking and potentially lifts up the manuscript’s value for the readership of the journal. Not to forget about the fact that many people from the dairy domain are unfamiliar with the horse kinematic studies using IMU technology or kinematics in general.

*We checked for discussion points lacking in the manuscript, but we feel all points are currently present. We feel that the kinematic IMU information is indeed valuable information in the dairy domain.*

Three points:
1) The change from “limb angle” to “distal limb angle” is a step in the right direction, however I think you could provide a good kinematic definition of what is exactly meant by this in the M&M section. Hence explain the relevance of the specific body segment’s angle and orientation (metatarsus and metacarpus) in the step cycle of the particular limb AND why it is useful to obtain this information. It could be added to the discussion from the perspective of the “clinical gait evaluation” in which multiple IMU could be used, opposed to the “farm setting” where a single IMU is mounted to one of the legs lameness screening, as you state to be the best location. However, single sided IMU’s make it more difficult to show asymmetry, hence a position in the neck might show the best trade-off, or not?

*The sagittal limb angle, i.e. protraction – retraction angle, is the most prominent movement of the limbs with the highest range of motion. It is useful to measure this limb angle because they are inertly related to the mechanism the animal uses to move (reverse pendulum model of the walk) and can indicate if the left and right limbs (as well as front and hind) are being used differently. From this angle, we can calculate temporal stride parameters such as stance, swing and all inter-limb related parameters. We have also demonstrated that these angles can be affected by lameness in horses at walk (Serra Braganca et al, 2020). How protraction/retraction are measured and defined varies significantly in the literature. We have chosen this angle as a practical approach because it’s a feasible manner to instrument the limb with a sensor on the metatarsal/-carpus and no substantial skin displacement occurs on this locations. Since, the angles we investigated (max and min) happen at the end of the swing and stance. Both of these events are closely related to the actual entire limb protraction/retraction angle which can be best measured closest to the claw. Using an IMU approach, there is no other solution to access these angles and if not using our approach, the entire limb kinematic could not be accessed.*

*We were was not aware of stating that the a single sided limb mounted IMU is the best location in regards to leg lameness screening. In our next paper, we will discuss this more deliberately and compare lameness data measured at all IMU locations before concluding which location is best to measure lameness.*

2) Whether it is the clinical or the farm setting, it cannot be neglected in the discussion that machine vision technologies are rapidly evolving to a situation which is easily compared with your technology. And, it is my personal opinion we need your type of studies to be able to explain/verify these new approaches, and vice versa. In addition, it is strange to me to read this opinion in the last line of your manuscript, without it being properly discussed.

*We agree that it is better to mention this also in the introduction of the manuscript. We have therefore added the sentence “This knowledge is paramount for the development of an automatic recognition method, which can also be used as an early warning system.” to line 72-73.*

3) If no more interpretation/aggregation of data is provided, basically it is left to the reader, then in general there are too many (vertical) displacement figures in my opinion.
a. One figure of all cows with one of the IMU’s is sufficient to show interindividual variations that is similar for all sensors.

*We agree that we show a lot of figures in the main text. We have therefore moved Fig 9 and 10, showing the vertical displacement of the withers and the back, to the supplementary materials. We have left the description in the manuscript as it is and only refer to the figures in the supplementary materials. Since the left and right tuber coxae show a different type of pattern compared to the other upper body locations, we would like to leave this figure in the main document. We also have moved Fig 12 and Fig 13, showing the vertical displacement of the head and collar, to the supplementary materials and only describe that these locations show a less consistent pattern.*

b. One cow’s all IMU’s to show the paths of various locations is enough to show the difference between locations.

*This is depicted in Fig 14.*

Minor:
Line 75 kinetic => kinematic
Other than that, no further comments.

*Thank you for noticing*

Reviewer #2: Thank you for this. The authors addressed all my comments.
see my previous comment (72-73: how the authors can explain the importance of applying the sensors in upper body in comparison to the "traditional" limb positions?)
I suggest to add your explanation to the text accordingly.

*We have added this explanation to the introduction in line 71-77: “Given the visibility of these body landmarks, even for a cow among herd mates, it would be of future interest to explore if asymmetries in the vertical excursions of the upper body could prove sensitive for detection of lame animals. This can be of particular interest in the development on automated lameness detection systems based on computer vision, where challenging sensor attachment is superfluous, but optical occlusion of limbs is a challenge. Prerequisite for such studies is the knowledge of normal walking gait.”*
